# The Effectiveness of Digital Cognitive Behavioral Therapy to Treat Insomnia Disorder in US Adults: Nationwide Decentralized Randomized Controlled Trial

**DOI:** 10.2196/84323

**Published:** 2025-12-04

**Authors:** Aric A Prather, Andrew D Krystal, Richard Emsley, Jenna Carl, Tali Ball, Kathryn Tarnai, Adrian Aguilera, Colin A Espie, Alasdair L Henry

**Affiliations:** 1 University of California, San Francisco San Francisco, CA United States; 2 King's College London London United Kingdom; 3 Big Health, Inc San Francisco, CA United States; 4 University of California, Berkeley Berkeley, CA United States; 5 University of Oxford Oxford United Kingdom

**Keywords:** insomnia, cognitive behavioral therapy, randomized controlled trial, digital mental health treatment, sleep

## Abstract

**Background:**

Cognitive behavioral therapy (CBT) is recommended as the first-line treatment for insomnia; however, few patients have access to it. A new class of Food and Drug Administration (FDA)–regulated digital CBT treatments has the potential to address this unmet need. These treatments are ordered or prescribed by health care providers and are fully automated, delivering CBT directly to patients without human coaches. This trial builds upon promising earlier digital cognitive behavioral therapy for insomnia (CBT-I) research by using a decentralized design to recruit a sample with greater representation of the US general population, including individuals from lower socioeconomic status groups who often face greater barriers to care.

**Objective:**

This decentralized trial evaluated the effectiveness of a fully automated digital CBT-I program (SleepioRx) for treating insomnia disorder compared with online sleep hygiene education (SHE) in a sample of participants recruited from across the United States.

**Methods:**

A decentralized, parallel-group randomized controlled trial was conducted between November 2022 and August 2023. Participants were recruited nationally from across the United States, and a total of 336 adults aged 22 and older, diagnosed with the fifth edition of the Diagnostic and Statistical Manual of Mental Disorders (DSM-5) insomnia disorder via structured clinical interview, were allocated 1:1 to either digital CBT-I (SleepioRx) or online SHE. The primary end points were insomnia severity, assessed using the Insomnia Severity Index (ISI), and sleep diary measures of sleep onset latency (SOL) and wake after sleep onset (WASO) at 10 weeks, with follow-up assessments at 16 and 24 weeks postrandomization.

**Results:**

Compared with SHE, SleepioRx showed statistically and clinically significant improvements on the ISI at posttreatment (10 weeks; Cohen d=0.60, *P*<.001), with effects sustained at follow-up (16 weeks; d=0.65, *P*<.001; and 24 weeks, d=0.77, *P*<.001). SleepioRx led to significant reductions in WASO at all time points (10 weeks, *P*=.003; 16 and 24 weeks, *P*<.001); however, effects on SOL were not statistically significant at an adjusted α (10 weeks, *P*=.01; 16 weeks, *P*=.07; 24 weeks, *P*=.27). SleepioRx participants had 2.5 times (odds ratio 2.52; *P*<.001, 99% CI 1.33-4.75) and 5.8 times (odds ratio 5.78; *P*<.001, 99% CI 2.11-15.84) greater odds of response and remission at week 10, respectively, with statistically and clinically significant differences in rates sustained at follow-up assessments (*P*<.001). SleepioRx also demonstrated sustained improvements in secondary sleep and broader mental health outcomes.

**Conclusions:**

The results of this trial demonstrate the effectiveness of digital CBT-I (SleepioRx) for treating insomnia, with gains sustained at 6 months, and support the FDA authorization of SleepioRx for the treatment of insomnia disorder. These findings underscore the potential of a new class of FDA-authorized, fully automated digital treatments to provide first-line, guideline-recommended CBT at scale. Efforts should now focus on expanding access to these evidence-based treatments.

**Trial Registration:**

ClinicalTrials.gov NCT05541055; https://clinicaltrials.gov/ct2/show/NCT05541055

## Introduction

Insomnia disorder is common, affecting approximately 10%-20% of the general population [1]. It is characterized by difficulties initiating sleep, maintaining sleep, or early morning awakening with an inability to resume sleep, occurring at least three nights per week for a period of at least three months and associated with significant daytime impairment [2,3]. The impacts of insomnia are considerable, with patients reporting substantial reductions in quality of life [4] and experiencing elevated risk of depression [5], anxiety [5], suicide [6], and cardiometabolic disease [7,8] compared with healthy sleeping counterparts.

Cognitive behavioral therapy (CBT) is universally recommended as the first-line treatment for insomnia disorder by national and international bodies [9-12]. Cognitive behavioral therapy for insomnia (CBT-I) treats insomnia by targeting psychological (eg, sleep-related worries, unhelpful beliefs about insomnia) and behavioral factors (eg, sleep-incompatible behaviors) that contribute to and maintain chronic sleep difficulties [13]. Typically, CBT-I incorporates a set of core interventions, including cognitive-focused techniques such as cognitive restructuring, cognitive control, and paradoxical intention, which help address dysfunctional beliefs and sleep-related worry and anxiety. To address behavioral factors, CBT-I includes stimulus control to strengthen the bed/bedroom environment as a cue for sleep, and sleep restriction, which helps consolidate sleep, regularize sleep-wake timing, and strengthen homeostatic sleep pressure. Additional techniques such as sleep hygiene education (SHE) and relaxation are included to support psychoeducation, improve lifestyle and environmental factors impacting sleep, and reduce somatic and cognitive arousal that interfere with sleep, respectively [13,14].

Despite strong recommendations for CBT-I as the first-line treatment, fewer than 10% of patients with insomnia receive CBT, largely due to systemic barriers, including a limited number of trained therapists and poor geographical distribution of providers, which restrict large-scale delivery [15-17]. These barriers to care are more pronounced in racial and ethnic minority groups [18-20]. In practice, insomnia is typically managed using pharmacotherapy [21-23], namely Food and Drug Administration (FDA)-approved medications (eg, zolpidem) or medications prescribed off-label (eg, trazodone), all of which can cause harmful side effects and are associated with dependency.

A proposed solution to address limited access to CBT is to deliver treatment digitally [24,25]. Substantial evidence supports the effectiveness of digital CBT for insomnia (digital CBT-I), demonstrating robust effects on insomnia and sleep outcomes compared with a range of controls [26-29]. Digital CBT-I has gained traction in recent years, with 2 digital CBT devices now cleared by the FDA for the treatment of insomnia disorder. One of these devices, SleepioRx, has been evaluated in 16 published randomized controlled trials (RCTs) [28,30-44], demonstrating clinically and statistically significant improvements in key insomnia outcomes, including insomnia severity and symptoms, sleep onset latency (SOL), and wake after sleep onset (WASO), compared with a range of comparators.

Although data robustly support the efficacy of digital CBT-I, several gaps in our knowledge remain. First, many digital clinical trials fail to enroll participants from a broad range of demographic groups, limiting the generalizability of the effects [45]. This is a common problem across clinical trials [46], and certain trial designs, such as decentralized trials, may help address participation barriers by relying on remote data collection methods [47].

Second, few trials have enrolled participants with a confirmed insomnia diagnosis established through structured clinical interviews. Previous trials have largely relied on validated patient-reported assessments for detecting insomnia; however, when digital CBT-I is used in practice, it may be prescribed based on a clinician’s interview.

To address these gaps, we conducted a decentralized RCT of SleepioRx, a fully automated digital CBT-I treatment, versus an online SHE control in participants diagnosed with insomnia disorder confirmed by structured clinical interviews and recruited nationally across the United States.

## Methods

### Study Design and Participants

The Clinical Effectiveness of Digital Insomnia Therapy (CrEDIT) trial was a decentralized, 2-arm, investigator-blind and participant-blind-to-hypothesis, parallel-group, superiority RCT of digital CBT (SleepioRx) versus online SHE. Participants aged 22 and older were recruited from across the United States via social media and were eligible to participate if they (1) met diagnostic criteria for insomnia disorder, confirmed using the Structured Clinical Interview for the Diagnostic and Statistical Manual of Mental Disorders (fifth edition)—Sleep Disorders Module (DSM-5; SCID; [48] interrater reliability for insomnia disorder: κ=1.0 [49]); (2) scored 16 or less on the 8-item Sleep Condition Indicator (SCI-8 [50]); (3) self-reported over 30 minutes SOL or WASO [51], or both, assessed via self-report; (4) resided in the United States; (5) understood oral and written English; and (6) were able and willing to comply with the protocol and provide informed consent. Individuals were excluded if (1) they self-reported currently receiving, expecting to start CBT for insomnia during study participation, or having received CBT for insomnia in the past 12 months; (2) if taking psychoactive medication, the dose was not stable for 5 or more half-lives; (3) they had a past or present diagnosis of psychosis, schizophrenia, or bipolar disorder, assessed via self-report and confirmed using the Mini International Neuropsychiatric Interview (MINI) for DSM-5 [52], or a seizure disorder; (4) they had an occupation requiring alertness/caution to avoid accidents (eg, long-haul driver, heavy machine operator, air traffic controller); (5) they self-reported an uncorrected hearing or vision impairment; (6) they had intellectual disability, or any neurocognitive or developmental disorder that would impede study participation, based on clinical judgment and adjudication by the principal investigator; and (7) they had any other condition that, in the opinion of the investigator, would make study participation not in the best interest of the participant. Following randomization, there were no restrictions on usual care for either arm. Consequently, the trial was, in effect, a comparison of digital CBT for insomnia + usual care versus SHE + usual care. The CrEDIT trial was prospectively registered (NCT05541055; first registered September 15, 2022).

### Ethics Considerations

The University of California, San Francisco (UCSF) Institutional Review Board (IRB) approved this study (IRB approval number 20-31243) and prospectively registered (NCT05541055). All potential participants completed a video call with trained staff to provide informed consent, which was collected electronically. Study participants were compensated commensurate with their time completing assessments, totaling up to US $190. Participants were instructed to contact study personnel if they experienced an adverse event, which would be evaluated and reviewed by the principal investigator and categorized and reported in accordance with ISO 14155:2020 [53] and UCSF procedures. No adverse events were reported by participants. All participant data were deidentified.

### Procedures

In accordance with a decentralized design, all study activities took place online. Recruitment ads were placed on social media platforms and shown nationwide across the United States, with the racial diversity and geographic distribution of recruitment monitored and adjusted to enhance the representativeness and socioeconomic and racial diversity of the sample. This was achieved by tracking the demographic characteristics of participants entering the trial and comparing them with US census data on demographics and socioeconomic status, as well as established benchmarks for the prevalence of insomnia across age and gender. Adjustments were made throughout the recruitment process to target groups and regions that are typically underrepresented in clinical trials or not adequately represented in the enrolled sample.

After providing initial consent and completing an online eligibility screener, potential participants were asked to complete a daily sleep diary for 10 days. Those who completed 7 or more diaries were invited to a video call interview with a study coordinator, during which full informed consent was obtained, the SCID and MINI were administered, and final eligibility for the trial was confirmed. Participants subsequently completed baseline assessments and were randomly assigned to receive either digital CBT-I (SleepioRx) or online SHE. Outcomes were assessed at 10, 16, and 24 weeks postrandomization. At each time point, assessments included self-reported outcomes, 10 days of sleep diaries, and a video call during which the SCID was administered. All outcome data were recorded using an online electronic data capture system independent of SleepioRx. Automated reminders were built into the system to follow-up with participants if outcome assessments were not completed within 3 days. To reduce the likelihood of fraudulent participant entries, participants were required to provide a valid form of photo identification during the initial video call assessment and briefly turn on their video so their likeness could be compared with the ID provided. Participants’ identity was reconfirmed at each subsequent video assessment using the same approach. Additionally, each participant was assigned a unique ID number, which they had to enter before completing any online assessments within the electronic data capture system and provide at the start of each follow-up video call, offering another means of participant verification. The study team followed a standardized risk and escalation protocol for participants reporting suicidality or risk of harm to others. All staff were trained and supervised by licensed clinicians, including psychologists and psychiatrists. When risk was reported, personnel conducted a formal assessment and, as appropriate, arranged psychological referrals or contacted emergency services.

Routine monitoring of data collected within the electronic data capture system was conducted throughout the trial. This included data reviews every 4-6 weeks by an independent data monitor, in accordance with a predefined trial monitoring plan, to ensure data completeness and quality. Any data issues identified were raised with the principal investigator and addressed. The study was also overseen by a Data Safety and Monitoring Board that met every 6 months and reviewed blinded trial data.

### Interventions

Participants allocated to the digital CBT-I arm received SleepioRx (Big Health Inc). SleepioRx is an FDA-cleared digital CBT-I intervention for the treatment of insomnia disorder that can be accessed on the order of a licensed health care provider (K233577; FDA, 2024 [54]). The program delivers cognitive (eg, cognitive restructuring and paradoxical intention), behavioral (eg, stimulus control, sleep restriction, and sleep hygiene), and physiological (eg, progressive muscle relaxation) techniques. SleepioRx techniques are delivered through audio, visual, and interactive elements, without any human coaching or assistance. Patient experience is tailored based on interactive features within the treatment, as well as daily sleep diaries. In this study, participants could access SleepioRx for up to 1 year.

Participants allocated to SHE received sleep hygiene advice representative of what patients would receive in routine clinical practice. This advice was based on resources developed by the American Academy of Sleep Medicine (AASM) [55], the National Sleep Foundation [56], and the National Heart, Lung, and Blood Institute [57]. Specifically, the sleep hygiene advice included psychoeducation on the links between sleep and health; factors to avoid, particularly in the evening, to improve sleep (eg, alcohol, nicotine, heavy meals, caffeine, daytime napping, late-night exercise); and tips for creating a healthy wind-down routine and a sleep-friendly sleep environment. This content also mirrors information on lifestyle, bedroom, and wind-down routines provided in SleepioRx. Consistent with real-world SHE, participants received all content at once and were encouraged to revisit it throughout the trial.

### Outcomes

The primary end points included the AASM-recommended gold-standard insomnia outcome measure, the Insomnia Severity Index (ISI) [58], as well as sleep diary–based SOL and WASO at 10 weeks postrandomization. Secondary outcomes were rates of response (change of ≥6) [59] and remission (score <8) [60] on the ISI, and self-reported insomnia symptoms assessed using the SCI-8 [50]. Rater-assessed remission of insomnia disorder, operationalized as no longer meeting diagnostic criteria on the SCID, was evaluated as an unpowered exploratory outcome. The ISI threshold for response was based on the established minimum clinically important difference [59], and the threshold for remission was based on previous research showing sensitivity and specificity above 90% in clinical samples [60].

Additional secondary outcomes included anxiety (7-item Generalized Anxiety Disorder scale [GAD-7] [61]), depressive symptoms (8-item Patient Health Questionnaire [PHQ-8] [62]), and sleep diary measures of total sleep time, total wake time, time in bed, sleep efficiency, and sleep quality. The use of concomitant treatment was recorded at baseline and at all outcome time points.

### Randomization and Masking

Participants were randomly assigned centrally within the electronic data capture platform (1:1) to SleepioRx or SHE using block randomization with block sizes of 6. No study personnel had access to the randomization sequence. All study personnel were blinded to participant allocation, and participants were blind to the study hypotheses. Both interventions were described as “sleep programs” in all participant-facing materials, and the details provided for each arm did not reveal which was the intervention or control. Furthermore, no indication of the directionality of the study hypotheses was provided.

### Statistical Analysis

Allowing for 30% attrition, a minimum of 332 participants (166 per arm) were required to detect a between-group effect of *d*=0.3 with 90% power. As recommended, a correction for 5 multiple comparisons (3 coprimary outcomes [ISI, SOL, and WASO] and 2 secondary outcomes [ISI response and ISI remission]) using a conservative Bonferroni approach yielded an α of .01 [63].

Primary analyses followed intention-to-treat (ITT) principles. Primary outcomes (ISI, SOL, and WASO) were analyzed using linear mixed-effects models, with baseline values, categorical time, randomized group, and a time × group interaction included as fixed effects, and participant as a random effect. Continuous secondary outcomes were analyzed using the same method. For all continuous outcomes, between-group Cohen *d* values were calculated based on the adjusted between-group difference divided by the pooled baseline SD for the full sample on that measure. Binary secondary outcomes (ISI response and remission and SCID remission) were analyzed using logistic mixed models, and odds ratios, CIs, and 2-sided *P* values are reported. One model per outcome was used to estimate effects at weeks 10 and 16, and effects at 24 weeks were assessed using separate models that incorporated the week 10, 16, and 24 outcomes. Clinical significance was prespecified as a between-group *d*≥0.5 for the ISI and a 10% or greater absolute difference in ISI response and remission rates between groups, per AASM criteria [9].

In addition to the primary ITT analyses, prespecified complier-average causal effect (CACE) and per-protocol analyses of the primary end points were conducted to assess the impact of adherence to digital CBT-I. CACE analyses compare the average outcome of those in the digital CBT-I arm who meet a compliance definition with the average outcome of the latent subgroup of individuals in the control group who *would have* met the compliance definition had they been randomized to the treatment (ie, a hidden counterfactual). Per-protocol analyses differ in that they compare the average outcome of all participants in the control group with the average outcome of only those in the treatment arm who meet the compliance definition. Per-protocol analyses therefore provide a biased estimate of treatment effect, whereas CACE analyses are randomization-respecting [64]. Three compliance definitions were used for both CACE and per-protocol analyses: (1) completion of at least one lesson; (2) completion of at least three lessons; and (3) completion of all 6 lessons. All analyses were conducted using Stata version 18.0 (StataCorp).

Linear mixed-effects models used to analyze continuous outcomes rely on a missing-at-random assumption. This was tested by examining differential predictors of missing outcomes based on baseline demographic and clinical factors; none were identified, and therefore no further statistical adjustment or imputation was applied in these models. The impact of missingness on binary outcome data was evaluated using a post hoc worst plausible case analysis (Multimedia Appendices 1 and 2) [65].

## Results

### Participant Recruitment, Baseline Characteristics, and Randomization

**Figure 1:**
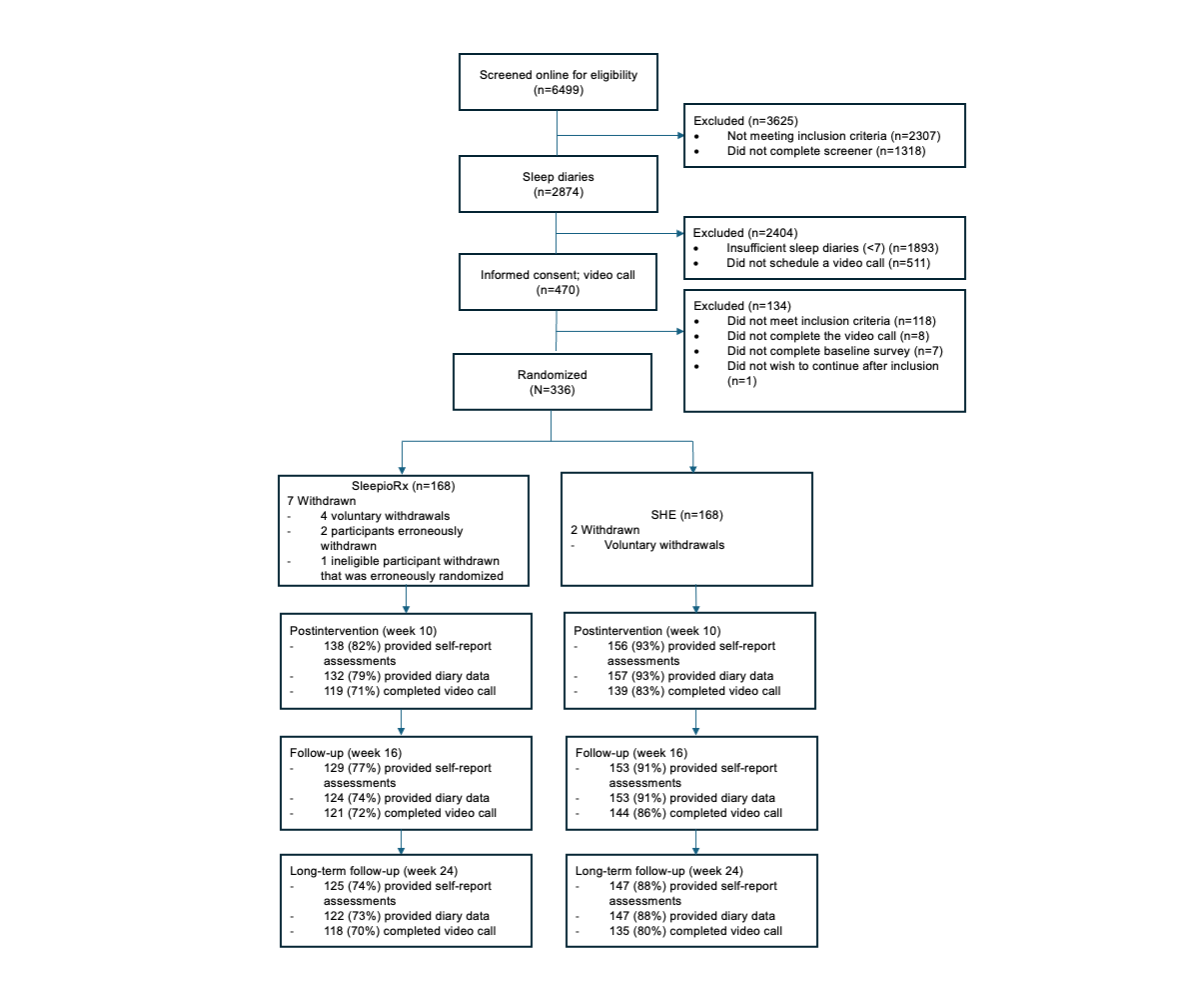
CONSORT (Consolidated Standards of Reporting Trials) flowchart. SHE: sleep hygiene education.

Participants were recruited between November 2022 and February 2023. Of the 6499 individuals who started the online screener, 470 progressed to the video call eligibility assessment, and a total of 336 participants were randomized to either digital CBT (n=168) or SHE (n=168). See Figure 1 for the study flow (and Multimedia Appendix 3 for the CONSORT checklist). As shown in Table 1, baseline characteristics were similar across groups. Participants demonstrated clinical levels of insomnia severity at baseline, with a mean ISI score of 18.23 (SD 3.96). The mean diary-assessed SOL was 54.11 (SD 40.10) minutes, and WASO was 47.38 (SD 42.15) minutes. On the SCI-8, participants had a mean score of 7.92 (SD 3.91), further indicating clinically severe impairment. Of the 168 participants allocated to SHE, 165 (98.2%) initiated treatment, and of those allocated to SleepioRx, 124 (73.8%) initiated treatment.

**Table 1:** Characteristics of the study sample.

Characteristic			Sleep hygiene education (n=168)		SleepioRx (n=168)		Sample (N=336)
Age (years), mean (SD)			46.84 (9.72)		45.96 (10.09)		46.40 (9.90)
**Gender, n (%)**							
	Women	90 (53.6)		98 (58.3)		188 (56.0)	
	Men	75 (44.6)		66 (39.3)		141 (42.0)	
	Transgender	1 (0.6)		1 (0.6)		2 (0.6)	
	Nonbinary	2 (1.2)		2 (1.2)		4 (1.2)	
	Other	0 (0)		1 (0.6)		1 (0.3)	
**Sex at birth, n (%)**							
	Female	93 (55.4)		102 (60.7)		195 (58.0)	
	Male	75 (44.6)		66 (39.3)		141 (42.0)	
**Race/ethnicity, n (%)**							
	Asian	11 (6.5)		7 (4.2)		18 (5.4)	
	Black	13 (7.7)		19 (11.3)		32 (9.5)	
	Latinx/Hispanic	12 (7.1)		11 (6.4)		23 (6.8)	
	Multiracial	11 (6.5)		8 (4.8)		19 (5.7)	
	Middle Eastern/North African	1 (0.6)		0 (0)		1 (0.3)	
	Native American/American Indian/Alaska Native/Indigenous	1 (0.6)		1 (0.6)		2 (0.6)	
	Pacific Islander/Native Hawaiian	0 (0)		0 (0)		0 (0)	
	White	119 (70.8)		121 (72.0)		240 (71.4)	
	Not specified	0 (0)		1 (0.6)		1 (0.3)	
**Employment, n (%)**							
	Full-time employed	79 (47.0)		83 (49.4)		162 (48.2)	
	Part-time employed	21 (12.5)		22 (13.1)		43 (12.8)	
	Unemployed	22 (13.1)		24 (14.3)		46 (13.7)	
	Retired	18 (10/7)		15 (8.9)		33 (9.8)	
	Full-time student	6 (3.6)		6 (3.6)		12 (3.6)	
	Full-time homemaker or carer	22 (13.1)		18 (10.7)		40 (11.9)	
**Education level, n (%)**							
	No formal qualifications	1 (0.6)		2 (1.2)		3 (0.9)	
	Secondary school/high school graduate	15 (8.9)		11 (6.5)		26 (7.7)	
	Some college	55 (32.7)		65 (38.7)		120 (35.7)	
	Undergraduate/bachelor’s degree	58 (34.5)		58 (34.5)		116 (34.5)	
	Postgraduate or professional degree	39 (23.2)		32 (19.0)		71 (21.1)	
**Marital status, n (%)**							
	Married	81 (48.2)		75 (44.6)		156 (46.4)	
	Divorced/separated	34 (20.2)		26 (15.5)		60 (17.9)	
	Never married	36 (21.4)		51 (30.4)		87 (25.9)	
	Partnered	12 (7.1)		12 (7.1)		24 (7.1)	
	Widowed	3 (1.8)		4 (2.4)		7 (2.1)	
	Prefer not to say	2 (1.2)		0 (0)		2 (0.6)	
**Household income (US $), n (%)**							
	Under 15,000	19 (11.3)		18 (10.7)		37 (11.0)	
	15,000 to 24,999	16 (9.5)		19 (11.3)		35 (10.4)	
	25,000 to 49,999	40 (23.8)		29 (17.3)		69 (20.5)	
	50,000 to 74,000	27 (16.1)		37 (22.0)		64 (19.0)	
	75,000 to 99,999	18 (10.7)		24 (14.3)		42 (12.5)	
	100,000 to 149,999	27 (16.1)		24 (14.3)		51 (15.2)	
	150,000 to 199,999	8 (4.8)		11 (6.5)		19 (5.7)	
	200,000 and over	13 (7.7)		6 (3.6)		19 (5.7)	
**Timezone, n (%)**							
	Eastern	80 (47.6)		91 (54.2)		171 (50.9)	
	Central	46 (27.4)		48 (28.6)		94 (28.0)	
	Mountain	16 (9.5)		12 (7.1)		28 (8.3)	
	Pacific	26 (15.5)		17 (10.1)		43 (12.8)	
	Alaska	0 (0)		0 (0)		0 (0)	
	Hawaii-Aleutian	0 (0)		0 (0)		0 (0)	
**Comorbidities** ^a^ **, n (%)**							
	Heart disease or high blood pressure	29 (17.3)		29 (17.3)		58 (17.3)	
	Diabetes	21 (12.5)		22 (13.1)		43 (12.8)	
	Stroke or other neurological problems	3 (1.8)		3 (1.8)		6 (1.8)	
	Cancer	7 (4.2)		8 (4.8)		15 (4.5)	
	Arthritis or other joint problems	36 (21.4)		51 (30.4)		87 (25.9)	
	Respiratory conditions (such as asthma, chronic obstructive pulmonary disease)	15 (8.9)		22 (13.1)		37 (11.0)	
	Digestive disorders (such as ulcers, irritable bowel syndrome, Crohn disease)	18 (10.7)		22 (13.1)		40 (11.9)	
	Depression	53 (31.5)		53 (31.5)		106 (31.5)	
	Anxiety	44 (26.2)		61 (36.3)		105 (31.3)	
	Hormonal problems	6 (3.6)		6 (3.6)		12 (3.6)	
**Dermatological conditions**			13 (7.7)		12 (7.1)		25 (7.4)
	None	55 (32.7)		50 (29.8)		105 (31.3)	
	Other	20 (11.9)		27 (16.1)		47 (14.0)	
**Use of prescription sleep medication, n (%)**							
	Yes	29 (17.3)		26 (15.5)		55 (16.4)	
	No	139 (82.7)		142 (84.5)		281 (83.6)	
**Use of over-the-counter sleep medication, n (%)**							
	Yes	54 (32.1)		63 (37.5)		117 (34.8)	
	No	114 (67.9)		105 (62.5)		219 (65.2)	
**Use of** ***any*** **sleep medication, n (%)**							
	Yes	69 (41.1)		77 (45.8)		146 (43.5)	
	No	99 (58.9)		91 (54.2)		190 (56.5)	
**Use of other prescription medication, n (%)**							
	Yes	85 (50.6)		83 (49.4)		168 (50.0)	
	No	83 (49.4)		85 (50.6)		168 (50.0)	
Insomnia Severity Index, mean (SD)			18.33 (4.19)		18.12 (3.72)		18.23 (3.96)
Wake after sleep onset, mean (SD)			46.06 (31.45)		48.71 (50.71)		47.38 (42.15)
Sleep onset latency, mean (SD)			53.92 (41.83)		54.30 (38.41)		54.11 (40.10)
8-Item Sleep Condition Indicator, mean (SD)			7.98 (3.91)		7.85 (3.93)		7.92 (3.91)
8-Item Patient Health Questionnaire, mean (SD)			9.84 (5.04)		9.52 (4.48)		9.68 (4.76)
7-Item Generalized Anxiety Disorder, mean (SD)			6.45 (5.01)		6.12 (4.56)		6.29 (4.79)
Total time in bed (minutes), mean (SD)			570.17 (122.21)		569.40 (110.24)		569.79 (116.20)
Total sleep time (minutes), mean (SD)			434.57 (132.44)		419.66 (114.11)		427.12 (123.65)
Total wake time (minutes), mean (SD)			178.91 (92.69)		185.69 (107.27)		182.30 (100.15)
Sleep efficiency (%), mean (SD)			70.66 (11.71)		69.21 (14.13)		69.93 (12.98)
Sleep quality, mean (SD)			2.49 (0.63)		2.48 (0.62)		2.48 (0.62)

^a^Not mutually exclusive categories.

### Treatment Effects on Primary Outcomes

Compared with SHE, SleepioRx produced statistically and clinically significant reductions in insomnia severity (ISI) at week 10 (*d*=0.60; *P*<.001), with maintenance of gains through weeks 16 (*d*=0.65) and 24 (*d*=0.77; Table 2). SleepioRx also resulted in a significant reduction in SOL at week 10 at an unadjusted α; however, this effect was no longer significant (*P*=.01)after applying the Bonferroni correction α. At weeks 16 and 24, the between-group differences in SOL were not statistically significant (week 16, *P=*.07; week 24, *P*=.27). SleepioRx led to statistically significant reductions in WASO at week 10 (*d*=0.21; *P*=.003), which were maintained throughout the follow-up period (week 16, *d*=0.28; week 24, *d*=0.29).

**Table 2:** ISI^a^, SOL^b^, and WASO^c^ summary statistics by group and time, and estimated treatment effects at week 10 (primary outcome), week 16 (follow-up), and week 24 (long-term follow-up). Adjusted differences are between-group mean differences.

Assessment		Unadjusted, mean (SD); n		Adjusted difference (SE)	99% CI	*P* value^d^	Cohen *d*
		Sleep hygiene education	SleepioRx				
**ISI**							
	Baseline	18.33 (4.19); 168	18.12 (3.72); 168	N/A^e^	N/A	N/A	N/A
	Week 10	14.74 (5.00); 156	12.11 (6.10); 138	–2.37 (0.56)	–3.81 to –0.92	<.001	0.60
	Week 16	14.60 (5.19); 153	11.37 (5.67); 129	–2.55 (0.57)	–4.01 to –1.09	<.001	0.65
	Week 24	14.80 (5.49); 147	11.00 (5.93); 125	–3.05 (0.59)	–4.56 to –1.54	<.001	0.77
**SOL**							
	Baseline	53.92 (41.83); 168	54.30 (38.41); 168	N/A	N/A	N/A	N/A
	Week 10	45.54 (48.82); 157	36.75 (32.00); 132	–9.14 (3.63)	–18.49 to 0.20	.01	0.23
	Week 16	38.82 (38.23); 153	32.56 (29.70); 124	–6.68 (3.69)	–16.18 to 2.83	.07	0.17
	Week 24	36.49 (40.73); 147	32.80 (43.59); 122	–4.37 (3.99)	–14.64 to 5.90	.27	0.11
**WASO**							
	Baseline	46.06 (31.45); 168	48.71 (50.71); 168	N/A	N/A	N/A	N/A
	Week 10	33.82 (30.06); 157	24.54 (21.40); 132	–8.86 (2.94)	–16.42 to –1.29	.003	0.21
	Week 16	35.27 (32.75); 153	23.97 (23.32); 124	–11.69 (2.97)	–19.35 to –4.03	<.001	0.28
	Week 24	35.64 (35.10); 147	24.11 (27.03); 122	–12.02 (3.19)	–20.24 to –3.80	<.001	0.29

^a^ISI: Insomnia Severity Index.

^b^SOL: Sleep onset latency.

^c^WASO: wake after sleep onset.

^d^*P*<.01 indicates statistical significance due to correction for multiple testing.

^e^N/A: not applicable.

### Treatment Effects on Secondary Outcomes

Response and remission based on the ISI were powered secondary outcomes. At week 10, SleepioRx participants had almost 6 times greater odds of ISI remission (ISI score <8) than control participants (odds ratio 5.78; *P*<.001; 99% CI 2.11-15.84; Table 3). Higher remission rates in the SleepioRx group compared with SHE were maintained at weeks 16 and 24. Similarly, participants randomized to SleepioRx had significantly greater odds of insomnia response (ISI score reduction ≥6) compared with SHE (Table 3). Across all time points, SleepioRx showed clinically significant absolute differences in response and remission rates relative to SHE (absolute between-group difference ≥10%).

**Table 3:** Analysis of ISI^a^ remission and response and SCID^b^ remission.

Analysis				Sleep hygiene education, n/N (%)		SleepioRx, n/N (%)		Odds ratio^c^; *P* value (99% CI)
**ISI remission (ISI score <8)**								
	**Week 10**						5.78; <.001 (2.11-15.84)	
		No	146/156 (93.6)		101/138 (73.2)			
		Yes	10/156 (6.4)		37/138 (26.8)			
	**Week 16**						3.49; <.001 (1.40-8.69)	
		No	139/153 (90.8)		95/129 (73.6)			
		Yes	14/153 (9.2)		34/129 (26.4)			
	**Week 24**						5.00; <.001 (1.92-13.03)	
		No	136/147 (92.5)		88/125 (70.4)			
		Yes	11/147 (7.5)		37/125 (29.6)			
**ISI response (ISI score reduction of ≥6)**								
	**Week 10**						2.52; <.001 (1.33-4.75)	
		No	110/156 (70.5)		68/138 (49.3)			
		Yes	46/156 (29.5)		70/138 (50.7)			
	**Week 16**						2.76; <.001 (1.43-5.35)	
		No	107/153 (69.9)		62/129 (48.1)			
		Yes	46/153 (30.1)		67/129 (51.9)			
	**Week 24**						2.92; <.001 (1.48-5.76)	
		No	96/147 (65.3)		54/125 (43.2)			
		Yes	51/147 (34.7)		71/125 (56.8)			
**SCID remission (defined as no longer meeting diagnostic criteria)**								
	**Week 10**						1.54; .09 (0.95-2.52)	
		No	78/139 (56.1)		54/119 (45.4)			
		Yes	61/139 (43.9)		65/119 (54.6)			
	**Week 16**						1.85; .02 (1.12-3.04)	
		No	70/144 (48.6)		41/121 (33.9)			
		Yes	74/144 (51.4)		80/121 (66.1)			
	**Week 24**						1.66; .05 (0.99-2.80)	
		No	57/135 (42.2)		36/118 (30.5)			
		Yes	78/135 (57.8)		82/118 (69.5)			

^a^ISI: Insomnia Severity Index.

^b^SCID: Structured Clinical Interview for the fifth edition of the Diagnostic and Statistical Manual of Mental Disorders.

^c^Odds ratio >1 for ISI means the odds of being in remission/response are higher in SleepioRx than in the control group.

Unpowered secondary outcomes included SCID remission, SCI-8, PHQ-8, GAD-7, and additional sleep diary measures. On SCID-based insomnia remission, clinically significant differences between groups were observed at all time points (absolute between-group difference ≥10%), with statistically significant differences detected only at week 16 (Table 3). For patient-reported symptoms of DSM-5 insomnia disorder (SCI-8), depression (PHQ-8), and anxiety (GAD-7), SleepioRx led to significantly greater improvements compared with control. Regarding secondary sleep diary measures, SleepioRx participants reported significantly better sleep efficiency and sleep quality at all follow-up time points compared with SHE participants. There were no consistent statistically significant differences between SleepioRx and SHE participants for other sleep diary outcomes, including total sleep time, time in bed, or total wake time (Table 4).

**Table 4:** SCI-8^a^, PHQ-8^b^, and GAD-7^c^ summary statistics by group and time, and estimated treatment effects. Effects are between-group mean differences.

Assessment			Unadjusted mean (SD); n				Adjusted difference (SE)		95% CI		*P* value^d^		Cohen *d*		
			Sleep hygiene education		SleepioRx										
**SCI-8**															
	Baseline	7.98 (3.91); 168		7.85 (3.93); 168		N/A^e^		N/A		N/A		N/A			
	Week 10	12.19 (5.49); 156		15.69 (6.88); 137		3.49 (0.67)		2.19 to 4.80		<.001		0.90			
	Week 16	12.68 (5.71); 153		16.06 (6.74); 129		3.24 (0.67)		1.92 to 4.56		<.001		0.83			
	Week 24	12.36 (6.09); 147		16.81 (7.47); 124		4.20 (0.70)		2.83 to 5.56		<.001		1.07			
**PHQ-8**															
	Baseline	9.84 (5.04); 168		9.52 (4.48); 168		N/A		N/A		N/A		N/A			
	Week 10	8.44 (4.89); 156		6.91 (5.39); 138		–1.03 (0.47)		–1.94 to –0.11		.03		0.22			
	Week 16	9.26 (5.51); 153		6.74 (5.08); 129		–1.61 (0.47)		–2.54 to –0.68		<.001		0.34			
	Week 24	9.05 (5.73); 147		6.56 (5.53); 125		–1.53 (0.49)		–2.50 to –0.56		.002		0.32			
**GAD-7**															
	Baseline	6.45 (5.01); 168		6.12 (4.56); 168		N/A		N/A		N/A		N/A			
	Week 10	6.81 (5.12); 156		5.42 (4.83); 137		-1.03 (0.46)		–1.92 to –0.14		.02		0.22			
	Week 16	7.03 (5.24); 153		5.57 (4.74); 129		–0.84 (0.46)		–1.75 to 0.06		.07		0.18			
	Week 24	7.14 (5.90); 147		5.18 (4.88); 125		1.28 (0.48)		–2.22 to –0.34		.008		0.27			
**Time in bed (minutes)**															
	Baseline	570.17 (122.21); 168		569.40 (110.24); 168		N/A		N/A		N/A		N/A			
	Week 10	554.96 (102.63; 157		567.06 (158.20); 132		17.35 (13.39)		–8.90 to 43.60		.19		0.15			
	Week 16	542.61 (106.45); 153		538.15 (115.59); 124		–2.46 (13.66)		–29.22 to 24.31		.86		0.02			
	Week 24	543.93 (99.00); 147		544.77 (105.75); 122		3.90 (13.03)		–21.65 to 29.44		.76		0.03			
**Total sleep time (minutes)**															
	Baseline	434.57 (132.44); 168		419.66 (114.11); 168		N/A		N/A		N/A		N/A			
	Week 10	449.48 (128.29); 157		471.45 (186.39); 132		28.97 (15.21)		–0.85 to 58.79		.06		0.23			
	Week 16	433.92 (116.92); 153		456.77 (116.62); 123		28.42 (15.52)		–2.00 to 58.83		.07		0.23			
	Week 24	446.89 (134.76); 147		456.48 (125.26); 122		19.04 (15.34)		–11.02 to 49.10		.21		0.15			
**Total wake time (minutes)**															
	Baseline	178.91 (92.69); 168		185.69 (107.27); 168		N/A		N/A		N/A		N/A			
	Week 10	146.60 (86.18); 157		133.90 (123.08); 129		–11.92 (11.41)		–34.28 to 10.43		.30		0.12			
	Week 16	144.37 (94.43); 153		119.70 (102.12); 124		–23.49 (11.56)		–46.15 to –0.83		.04		0.23			
	Week 24	128.97 (77.02); 146		118.54 (99.14); 122		–10.49 (11.10)		–32.25 to 11.26		.34		0.10			
**Sleep efficiency (0-100)**															
	Baseline	70.66 (11.71); 168		69.21 (14.13); 168		N/A		N/A		N/A		N/A			
	Week 10	75.33 (13.23); 157		78.04 (14.12); 129		2.95 (1.39)		0.21 to 5.68		.03		0.23			
	Week 16	75.59 (13.47); 153		80.02 (12.23); 123		4.31 (1.41)		1.54 to 7.08		.002		0.33			
	Week 24	77.11 (12.65); 146		79.71 (13.73); 122		2.83 (1.41)		0.07 to 5.59		.04		0.22			
**Sleep quality (0-5)**															
	Baseline	2.49 (0.63); 168		2.48 (0.62); 168		N/A		N/A		N/A		N/A			
	Week 10	2.86 (0.70); 155		3.09 (0.82); 129		0.25 (0.08)		0.09 to 0.41		.002		0.40			
	Week 16	2.83 (0.72); 155		3.14 (0.80); 127		0.29 (0.08)		0.13 to 0.45		<.001		0.46			
	Week 24	2.91 (0.78); 147		3.16 (0.82); 122		0.24 (0.08)		0.07 to 0.40		.005		0.38			

^a^SCI-8: 8-item Sleep Condition Indicator.

^b^PHQ-8: 8-item Patient Health Questionnaire.

^c^GAD-7: 7-item Generalized Anxiety Disorder.

^d^*P*<.05 indicates statistical significance due to no correction for multiple testing for these outcomes.

^e^N/A: not applicable.

### Impact of Adherence on Primary Outcomes

Per-protocol and CACE analyses of the primary end points were conducted to assess the impact of adherence to SleepioRx. Greater adherence was associated with larger improvements in ISI, SOL, and WASO scores compared with SHE. Additionally, participant demographics were largely consistent across all compliance measures (see Multimedia Appendices 4-7).

### Post Hoc Analyses

Initial analyses revealed that study participants, on average, reported at least seven hours of sleep, which is higher than in several other similar trials [33,66,67] and could indicate minimal room for improvement in SOL and WASO despite high overall insomnia severity. Therefore, we conducted post hoc analyses to evaluate the effects of SleepioRx on the primary end points in participants with a sleep duration of 6.5 hours or less. Treatment effects in this cohort were larger than those observed in the ITT sample (see Multimedia Appendix 8).

Further post hoc analyses were conducted on ISI response and remission outcomes to evaluate the robustness of effects to missing data under worst-plausible assumptions (see Multimedia Appendices 1 and 2). These analyses demonstrate that SleepioRx continues to produce statistically and clinically significant differences in rates of remission relative to control.

## Discussion

### Principal Findings

The primary goal of this study was to test the effectiveness of an FDA-regulated digital cognitive behavioral treatment for insomnia (SleepioRx) in a large, representative sample of adults diagnosed with insomnia disorder using a structured clinical interview. Consistent with our hypotheses, participants allocated to SleepioRx demonstrated significant and sustained improvements in insomnia symptoms compared with a SHE control. Individuals randomized to SleepioRx also showed improvements in diary-based measures compared with SHE, including fewer minutes awake after sleep onset and shorter SOL, although the latter was not statistically significant after conservative correction for multiple comparisons. In secondary analyses, SleepioRx also produced improvements in diary-assessed sleep efficiency and subjective sleep quality, as well as considerable and sustained improvements in symptoms of depression and anxiety compared with SHE. Our findings are consistent with the growing literature showing that digital CBT-I, including SleepioRx, can lead to significant reductions in co-occurring depressive and anxiety symptoms [5,68,69]. Given the substantial need for effective treatments to address insomnia symptoms, these data provide further support for the role of digital CBT-I in increasing access to guideline-recommended care.

To our knowledge, this is the first study to investigate whether digital CBT-I predicts remission of insomnia disorder using structured clinical interviews. In an unpowered analysis, we found some evidence that participants randomized to SleepioRx were less likely to meet diagnostic criteria for insomnia disorder compared with the control condition, with clinically significant differences at all time points and statistical significance at the 16-week assessment. Findings were stronger when remission was assessed using the ISI remission criteria (ie, ISI score <8), exceeding the prespecified threshold for clinical significance, although absolute rates of remission were slightly lower than those reported in a recent meta-analysis (30% vs 41%) [70]. This difference may be due to the sample included in this study, which included a significant proportion of individuals from underserved backgrounds who may experience greater barriers to care that impact treatment outcomes (eg, [19]). Remission rates using the SCID were high in both the SleepioRx and SHE arms, with smaller between-group differences than expected given the effect sizes in other outcome measures. This may be attributable to the binary nature of the SCID in establishing disorder presence versus absence. Additionally, regression to the mean, which is often a concern in clinical trials [71], may have had a greater impact on the SCID outcome compared with other insomnia measures. Unlike instruments such as the ISI and SCI-8, remission status on the SCID is determined if a participant fails to meet even 1 diagnostic criterion (eg, reduction to fewer than 3 nights per week of disturbance over the past 3 months). Future work should further explore the utility of using diagnostic criteria to establish insomnia remission in a suitably powered analysis. It is likely, however, that both patient-reported and clinician-reported measures could be used in tandem when evaluating outcomes.

### Strengths

A key strength of this study was the regional, racial, and socioeconomic breadth of the sample. Indeed, the demographic composition closely reflected the US population with regard to gender [58], educational attainment [72], income [73], and employment status [74]. Although the racial characteristics of the sample did not perfectly match the US population [75], it demonstrated greater racial diversity than observed in many other CBT trials (eg, [34,76]). The results demonstrate sustained benefits to sleep in a population of individuals likely experiencing greater life stressors and barriers to accessing and adhering to care (eg, [18,19]). Although other studies of SleepioRx have included more racially diverse samples (eg, [32]), these have been limited to single-site designs and have not recruited participants from across the United States. Furthermore, many other studies of digital CBT either include very homogeneous samples (eg, [36]) or fail to report details regarding participant demographics and socioeconomic characteristics. Future trials should build upon this work and evaluate whether differential treatment engagement or responses occur in appropriately powered subsample analyses.

Related to the above, the demographic inclusiveness of this study underscores the potential of decentralized trials for evaluating digital mental health treatments. The use of decentralized trials has proliferated in recent years, largely catalyzed by the COVID-19 pandemic [61]. This trial demonstrates that decentralized designs can be used effectively to assess the effectiveness of digital mental health treatments and that such designs may support greater inclusivity in trials [47].

A further strength of this study is the a priori multiplicity adjustment; nevertheless, Bonferroni corrections are known to be highly conservative [77] and may have increased the likelihood of type II errors. Had an alternative, less conservative method been used (eg, the Holm-Bonferroni procedure) [78], statistical significance would have been observed for all 5 powered outcomes in the ITT analyses.

### Limitations

Despite the many strengths of this study, several limitations should be noted. First, the study sample was recruited via social media and may not reflect the general population or patients seeking treatment through health care providers. Compared with other trials, participants reported longer sleep duration and lower SOL and WASO at baseline, despite meeting diagnostic criteria for insomnia disorder or chronic insomnia, which do not require a minimum SOL or WASO duration [2,3]. This may have limited the ability to detect improvements in SOL and WASO specifically, resulting in smaller-than-expected effects. In addition, our follow-up period ended at 24 weeks postrandomization, and we cannot infer benefits sustained beyond that point. That said, other studies support treatment benefits as far out as 3 years after starting digital CBT-I [79]. Finally, a slightly higher drop-out rate was observed in those randomized to the SleepioRx arm compared with SHE, although this is common in digital therapy studies, and results were robust to worst-case plausible assumptions regarding missing data [34,76].

This trial contributed to the evidence base supporting SleepioRx’s FDA clearance as a treatment for insomnia disorder and demonstrates the clinical utility and potential of software-based digital therapeutics for behavioral health [24]. In 2025, the Centers for Medicare & Medicaid Services (CMS) established a national policy and reimbursement codes for FDA-cleared digital mental health treatments. These treatments are ordered and overseen by licensed health care providers (eg, physicians, nurse practitioners, psychologists, social workers) and delivered directly to patients in an automated, high-fidelity format. Under this policy, providers and health systems procure such interventions from manufacturers and receive reimbursement for both the treatment cost and associated treatment management services, representing a significant advance in scalable access to evidence-based, first-line CBT-I.

### Conclusions

This decentralized RCT found that digital CBT-I (SleepioRx) was effective in treating insomnia disorder in a large national sample of adults compared with SHE. The data indicate that digital CBT-I is cost-effective relative to other treatments [80] and can be implemented at scale [81]. Further efforts should be made to increase the availability of evidence-based digital CBT-I to expand access to first-line treatment for insomnia disorder.

## Appendix

Multimedia Appendix 1 Worst-case plausible analyses demonstrating the robustness of Insomnia Severity Index remission results to missing data.

Multimedia Appendix 2 Worst-case plausible analyses demonstrating the robustness of Insomnia Severity Index response results to missing data.

Multimedia Appendix 3 CONSORT (Consolidated Standards of Reporting Trials) checklist.

Multimedia Appendix 4 Descriptive statistics by compliance group in the SleepioRx arm. Summary statistics are presented as mean and SD or number and percentages.

Multimedia Appendix 5

Multimedia Appendix 6

Multimedia Appendix 7

Multimedia Appendix 8 Summary statistics for Insomnia Severity Index, sleep onset latency, and wake after sleep onset by group and time, and estimated treatment effects at weeks 10, 16, and 24 among participants with self-reported sleep duration ≤6.5 hours. Effects represent between-group mean differences derived from prespecified linear mixed-effects model analyses.

## Abbreviations

AASM: American Academy of Sleep Medicine
CACE: complier-average causal effect
CBT: cognitive behavioral therapy
CBT-I: cognitive behavioral therapy for insomnia
CMS: Centers for Medicare & Medicaid Services
CrEDIT: Clinical Effectiveness of Digital Insomnia Therapy
DSM-5: fifth edition of the Diagnostic and Statistical Manual of Mental Disorders
FDA: Food and Drug Administration
GAD-7: 7-item Generalized Anxiety Disorder
IRB: institutional review board
ISI: Insomnia Severity Index
ITT: intention to treat
MINI: Mini International Neuropsychiatric Interview
NIHR: National Institute for Health and Care Research
PHQ-8: 8-item Patient Health Questionnaire
RCT: randomized controlled trial
SCI-8: 8-item Sleep Condition Indicator
SCID: Structured Clinical Interview for DSM-5
SHE: sleep hygiene education
SOL: sleep onset latency
UCSF: University of California, San Francisco
WASO: wake after sleep onset

## Data Availability

The datasets generated or analyzed during this current study are not publicly available, but are available from the corresponding author (AP) on reasonable request.
