# Supplementary material for: The Effectiveness of Digital Cognitive Behavioral Therapy to Treat Insomnia Disorder in US Adults: Nationwide Decentralized Randomized Controlled Trial
Source: JMIR Ment Health. 2025 Dec 4;12:e84323. doi: 10.2196/84323 (PMC12715469; doi:10.2196/84323)
Supplement: Multimedia Appendix 1 [file mental_v12i1e84323_app1.docx]

**Supplement Table 6:** Worst case plausible analyses showing the robustness of ISI remission results to missingness

| **Timepoint** | **ISI remission** | **SHE, n (%)** | **SleepioRx, n (%)** | **Difference in remission rates** | **Chi-sq** | ***p*-value** |
| --- | --- | --- | --- | --- | --- | --- |
| Week 10 | Yes | 11 (6) | 40 (24) | 17% | 19.44 | <0.001 |
|  | No | 157 (94) | 128 (76) |  |  |  |
| Week 24 | Yes | 13 (7) | 41 (25) | 17% | 17.30 | <0.001 |
|  | No | 155 (93) | 127 (75) |  |  |  |

Results represent the likelihood of achieving ISI remission for participants in the SleepioRx arm compared to the SHE arm, with missing data at each time point imputed following GRADE guidelines (Guyatt et al., 2017). Specifically, missing SHE arm data were assumed to follow the same remission rate as was observed in the SHE arm, and missing SleepioRx arm data were conservatively assumed to have a non-remission rate three times higher than the rate observed in SleepioRx arm.
