# Supplementary material for: The Effectiveness of Digital Cognitive Behavioral Therapy to Treat Insomnia Disorder in US Adults: Nationwide Decentralized Randomized Controlled Trial
Source: JMIR Ment Health. 2025 Dec 4;12:e84323. doi: 10.2196/84323 (PMC12715469; doi:10.2196/84323)
Supplement: Multimedia Appendix 4 [file mental_v12i1e84323_app4.docx]

**Supplement Table 1**: Descriptive statistics by compliance group in SleepioRx arm. Summary statistics are presented as mean and standard deviation, and/or number and percentages.

| **Characteristic** | **SleepioRx**  **N=168** | **Any lessons**  **N=124** | **≥ 3 lessons**  **N=89** | **All 6 lessons**  **N=77** |
| --- | --- | --- | --- | --- |
| Age, mean (SD),y | 45.96 (10.09) | 44.91 (10.05) | 45.21 (9.90) | 44.91 (9.77) |
| Gender, N (%) | | | | |
| Women | 98 (58) | 74 (60) | 53 (60) | 46 (60) |
| Men | 66 (39) | 47 (38) | 34 (38) | 29 (38) |
| Transgender | 1 (1) | 1 (1) | 1 (1) | 1 (1) |
| Non-binary | 2 (1) | 1 (1) | 0 | 0 |
| Other | 1 (1) | 1 (1) | 1 (1) | 1 (1) |
| Race/ethnicity, N (%) | | | | |
| Asian | 7 (4) | 6 (5) | 2 (2) | 2 (3) |
| Black | 19 (11) | 12 (10) | 7 (8) | 6 (8) |
| Latinx / Hispanic | 11 (7) | 9 (7) | 7 (8) | 5 (6) |
| Multiracial | 8 (5) | 6 (5) | 6 (7) | 5 (6) |
| Middle Eastern / North African | 0 | 0 | 0 | 0 |
| Native American / American Indian / Alaska Native / Indigenous | 1 (1) | 1 (1) | 0 | 0 |
| Pacific Islander / Native Hawaiian | 0 | 0 | 0 | 0 |
| White | 121 (72) | 90 (73) | 67 (75) | 59 (77) |
| Not specified | 1 (1) | 0 | 0 | 0 |
| Employment, N (%) | | | | |
| Full-time employed | 83 (49) | 66 (53) | 45 (51) | 41 (53) |
| Part-time employed | 22 (13) | 11 (9) | 6 (7) | 6 (8) |
| Unemployed | 24 (14) | 15 (12) | 13 (15) | 8 (10) |
| Retired | 15 (9) | 12 (10) | 10 (11) | 8 (10) |
| Full-time student | 6 (4) | 5 (4) | 3 (3) | 3 (4) |
| Full-time homemaker/carer | 18 (11) | 15 (12) | 12 (13) | 11 (14) |
| Education level, N (%) | | | | |
| No formal qualifications | 2 (1) | 1 (1) | 1 (1) | 1 (1) |
| Secondary school / high school graduate | 11 (7) | 8 (6) | 6 (7) | 5 (6) |
| Some college | 65 (39) | 48 (39) | 33 (37) | 26 (34) |
| Undergraduate / Bachelor’s degree | 58 (35) | 42 (34) | 30 (34) | 27 (35) |
| Postgraduate or professional degree | 32 (19) | 25 (20) | 19 (21) | 18 (23) |
| Marital status, N (%) | | | | |
| Married | 75 (45) | 59 (48) | 40 (45) | 36 (47) |
| Divorced/Separated | 26 (15) | 16 (13) | 10 (11) | 6 (8) |
| Never married | 51 (30) | 38 (31) | 31 (35) | 27 (35) |
| Partnered | 12 (7) | 9 (7) | 6 (7) | 6 (8) |
| Widowed | 4 (2) | 2 (2) | 2 (2) | 2 (3) |
| Prefer not to say | 0 | 0 | 0 | 0 |
| Household income, N (%) | | | | |
| Under 15,000 | 18 (11) | 12 (10) | 10 (11) | 7 (9) |
| 15,000 to 24,999 | 19 (11) | 14 (11) | 9 (10) | 6 (8) |
| 25,000 to 49,999 | 29 (17) | 22 (18) | 18 (20) | 15 (20) |
| 50,000 to 74,000 | 37 (22) | 28 (23) | 19 (21) | 17 (22) |
| 75,000 to 99,999 | 24 (14) | 17 (14) | 11 (12) | 11 (14) |
| 100,000 to 149,999 | 24 (14) | 18 (15) | 13 (15) | 12 (16) |
| 150,000 to 199,999 | 11 (7) | 8 (6) | 5 (6) | 5 (6) |
| 200,000 and over | 6 (4) | 5 (4) | 4 (4) | 4 (5) |
| Timezone, N (%) | | | | |
| Eastern | 91 (54) | 68 (55) | 48 (54) | 43 (56) |
| Central | 48 (29) | 33 (27) | 25 (28) | 21 (27) |
| Mountain | 12 (7) | 11 (9) | 9 (10) | 6 (8) |
| Pacific | 17 (10) | 12 (10) | 7 (8) | 7 (9) |
| Alaska | 0 | 0 | 0 | 0 |
| Hawaii-Aleutian | 0 | 0 | 0 | 0 |
| Comorbidities*, N (%) | | | | |
| Heart disease or high blood pressure | 29 (17) | 15 (12) | 12 (13) | 7 (9) |
| Diabetes | 22 (13) | 15 (12) | 8 (9) | 7 (9) |
| Stroke or other neurological problems | 3 (2) | 3 (2) | 1 (1) | 0 |
| Cancer | 8 (4) | 6 (5) | 5 (6) | 5 (6) |
| Arthritis or other joint problems | 51 (30) | 34 (27) | 26 (21) | 21 (27) |
| Respiratory conditions (such as asthma, COPD) | 22 (13) | 14 (11) | 9 (10) | 7 (9) |
| Digestive disorders (such as ulcers, IBS, Crohn’s disease) | 18 (11) | 14 (11) | 12 (13) | 9 (12) |
| Depression | 53 (32) | 38 (31) | 27 (30) | 19 (25) |
| Anxiety | 61 (36) | 45 (36) | 31 (35) | 23 (30) |
| Hormonal problems | 6 (4) | 3 (2) | 3 (3) | 2 (3) |
| Dermatological conditions | 12 (7) | 6 (5) | 4 (4) | 3 (4) |
| None | 50 (30) | 37 (30) | 25 (28) | 23 (30) |
| Other | 27 (16) | 18 (15) | 16 (18) | 14 (18) |
| Use of prescription sleep medication, N (%)  Yes  No | 26 (15)  142 (85) | 18 (14)  106 (85) | 13 (15)  76 (85) | 12 (16)  65 (84) |
| Use of over the counter sleep medication, N (%)  Yes  No | 63 (37)  105 (63) | 45 (36)  79 (64) | 34 (38)  55 (62) | 30 (39)  47 (61) |
| Use of ANY sleep medication, N (%)  Yes  No | 77 (46)  91 (54) | 55 (44)  69 (56) | 41 (46)  48 (54) | 36 (47)  41 (53) |
| Use of other prescription medication, N (%)  Yes  No | 83 (49)  85 (51) | 61 (49)  63 (51) | 46 (52)  43 (48) | 39 (51)  38 (49) |

*not mutually exclusive categories.
