# Supplementary material for: The Effectiveness of Digital Cognitive Behavioral Therapy to Treat Insomnia Disorder in US Adults: Nationwide Decentralized Randomized Controlled Trial
Source: JMIR Ment Health. 2025 Dec 4;12:e84323. doi: 10.2196/84323 (PMC12715469; doi:10.2196/84323)
Supplement: Multimedia Appendix 5 [file mental_v12i1e84323_app5.docx]

**Supplement Table 2**: Per-protocol and CACE analyses for the ISI. *p*<0.01 indicates statistical significance due to correction for multiple testing.

| **Compliance definition** | **Non-compliance analysis** | **Adjusted Difference (SE); p-value (99% CI)**  **Cohen’s *d*** | | |
| --- | --- | --- | --- | --- |
|  |  | **10 weeks** | **16 weeks** | **24 weeks** |
| **1 lesson complete** | Per-protocol | -3.29 (0.59); <0.001  (-4.80, -1.78)  0.83 | -3.71 (0.59); <0.001 (-5.22, -2.19)  0.94 | -3.86 (0.62); <0.001  (-5.45, -2.28)  0.98 |
|  | CACE | -3.33 (0.75); <0.001  (-5.27, -1.40)  0.84 | -3.61 (0.71); <0.001  (-5.45, -1.78)  0.91 | -4.35 (0.82); <0.001 (-6.47, -2.24)  1.10 |
| **3 or more lessons complete** | Per-protocol | -3.96 (0.63); <0.001 (-5.58, -2.35)  1.00 | -4.38 (0.63); <0.001 (-6.01, -2.75)  1.11 | -4.47 (0.66); <0.001 (-6.16, -2.77)  1.13 |
|  | CACE | -4.05 (0.90); <0.001  (-6.37, -1.73)  1.02 | -4.49 (0.88); <0.001  (-6.75, -2.24)  1.13 | -5.26 (0.99); <0.001 (-7.80, -2.71)  1.33 |
| **All 6 lessons complete** | Per-protocol | -4.61 (0.63); <0.001  (-6.24, -2.98)  1.16 | -4.90 (0.64); <0.001 (-6.54, -3.26)  1.24 | -5.13 (0.66); <0.001 (-6.84, -3.42)  1.30 |
|  | CACE | -4.48 (0.98); <0.001  (-6.99, -1.96)  1.13 | -4.92 (0.94); <0.001  (-7.36, -2.49)  1.24 | -5.77 (1.06); <0.001 (-8.51, -3.04)  1.46 |
