# Supplementary material for: The Effectiveness of Digital Cognitive Behavioral Therapy to Treat Insomnia Disorder in US Adults: Nationwide Decentralized Randomized Controlled Trial
Source: JMIR Ment Health. 2025 Dec 4;12:e84323. doi: 10.2196/84323 (PMC12715469; doi:10.2196/84323)
Supplement: Multimedia Appendix 6 [file mental_v12i1e84323_app6.docx]

**Supplement Table 3**: Per-protocol and CACE analyses for SOL. *p*<0.01 indicates statistical significance due to correction for multiple testing.

| **Compliance definition** | **Non-compliance analysis** | **Adjusted Difference (SE); p-value (99% CI)**  **Cohen’s *d*** | | |
| --- | --- | --- | --- | --- |
|  |  | **10 weeks** | **16 weeks** | **24 weeks** |
| **1 lesson complete** | Per-protocol | -12.28 (3.99); 0.002  (-22.56, -2.00)  0.31 | -7.43 (4.03); 0.065  (-17.80, 2.94)  0.19 | -5.56 (4.41); 0.207 (-16.92, 5.79)  0.14 |
|  | CACE | -12.30 (5.25); 0.019  (-25.81, 1.22)  0.31 | -9.18 (4.27); 0.032  (-20.19, 1.83)  0.23 | -6.16 (5.90); 0.296  (-21.36, 9.03)  0.15 |
| **3 or more lessons complete** | Per-protocol | -12.17 (4.34); 0.005  (-23.36, -0.99)  0.30 | -8.33 (4.40); 0.058  (-19.66, 2.99)  0.21 | -4.86 (4.83); 0.315 (-17.30, 7.59)  0.12 |
|  | CACE | -14.67 (6.29); 0.020  (-30.87, 1.54)  0.37 | -11.13 (5.18); 0.032  (-24.48, 2.22)  0.28 | -7.53 (7.22); 0.297 (-26.12, 11.07)  0.19 |
| **All 6 lessons complete** | Per-protocol | -15.59 (4.39); 0.001  (-26.88, -4.29)  0.39 | -11.20 (4.43); 0.011  (-22.61, 0.21)  0.28 | -8.12 (4.90); 0.098  (-20.76, 4.51)  0.20 |
|  | CACE | -16.23 (6.89); 0.019  (-33.98, 1.52)  0.40 | -12.20 (5.62); 0.030  (-26.69, 2.29)  0.30 | -8.28 (7.90); 0.295  (-28.64, 12.08)  0.21 |
