# Supplementary material for: The Effectiveness of Digital Cognitive Behavioral Therapy to Treat Insomnia Disorder in US Adults: Nationwide Decentralized Randomized Controlled Trial
Source: JMIR Ment Health. 2025 Dec 4;12:e84323. doi: 10.2196/84323 (PMC12715469; doi:10.2196/84323)
Supplement: Multimedia Appendix 7 [file mental_v12i1e84323_app7.docx]

**Supplement Table 4**: Per-protocol and CACE analyses for WASO. *p*<0.01 indicates statistical significance due to correction for multiple testing.

| **Compliance measure** | **Non-compliance analysis** | **Adjusted Difference (SE); p-value (99% CI)**  **Cohen’s *d*** | | |
| --- | --- | --- | --- | --- |
|  |  | **10 weeks** | **16 weeks** | **24 weeks** |
| **1 lesson complete** | Per-protocol | -9.30 (3.29); 0.005  (-17.77, -0.83)  0.22 | -12.52 (3.31); <0.001  (-21.04, -4.01)  0.30 | -12.77 (3.56); <0.001  (-21.95, -3.59)  0.30 |
|  | CACE | -12.76 (3.73); 0.001  (-22.37, -3.15)  0.30 | -14.23 (4.11); 0.001  (-24.83, -3.63)  0.34 | -14.48 (4.65); 0.002  (-26.45, -2.51)  0.34 |
| **3 or more lessons complete** | Per-protocol | -10.07 (3.56); 0.005  (-19.24, -0.91)  0.24 | -13.90 (3.59); <0.001  (-23.14, -4.66)  0.33 | -13.99 (3.87); <0.001  (-23.97, -4.01)  0.33 |
|  | CACE | -15.21 (4.47); 0.001  (-26.74, -3.69)  0.36 | -17.25 (5.00); 0.001  (-30.13, -4.38)  0.41 | -17.68 (5.69); 0.002  (-32.33, -3.03)  0.42 |
| **All 6 lessons complete** | Per-protocol | -10.01 (3.68); 0.007  (-19.49, -0.52)  0.24 | -13.93 (3.71); <0.001  (-23.47, -4.38)  0.33 | -14.96 (4.00); <0.001  (-25.27, -4.65)  0.35 |
|  | CACE | -16.83 (4.98); 0.001  (-29.66, -3.99)  0.40 | -18.90 (5.49); 0.001  (-33.05, -4.76)  0.45 | -19.44 (6.23); 0.002  (-35.49, -3.39)  0.46 |
