# Supplementary material for: The Effectiveness of Digital Cognitive Behavioral Therapy to Treat Insomnia Disorder in US Adults: Nationwide Decentralized Randomized Controlled Trial
Source: JMIR Ment Health. 2025 Dec 4;12:e84323. doi: 10.2196/84323 (PMC12715469; doi:10.2196/84323)
Supplement: Multimedia Appendix 8 [file mental_v12i1e84323_app8.docx]

**Supplement Table 5:**  ISI, SOL, and WASO summary statistics by group and time, and estimated treatment effects at week 10, week 16, and week 24 for participants with a self-reported sleep duration of ≤6.5 hours. Effects are between-group mean differences based on pre-specified linear mixed-effect model analysis methods.

| **Assessment** | **Unadjusted mean (SD); n** | | **Adjusted difference (SE)** | **95% CI** | ***p*-value** | **Cohen’s *d*** |
| --- | --- | --- | --- | --- | --- | --- |
|  | **SHE** | **Sleepio** |  |  |  |  |
| **ISI** | | | | | | |
| Baseline | 18.95 (4.38); 65 | 18.75 (3.85); 64 |  |  |  |  |
| Week 10 | 14.79 (4.85); 63 | 12.46 (6.41); 52 | -2.30 (0.94) | -4.13, -0.46 | 0.014 | 0.56 |
| Week 16 | 15.07 (5.42); 61 | 11.43 (5.72); 47 | -3.16 (0.95) | -5.03, -1.30 | 0.001 | 0.77 |
| Week 24 | 15.72 (5.66); 58 | 11.23 (6.23); 47 | -3.91 (0.98) | -5.84, -1.98 | <0.001 | 0.95 |
| **SOL** | | | | | | |
| Baseline | 63.35 (43.53); 65 | 62.73 (47.06); 64 |  |  |  |  |
| Week 10 | 61.36 (61.03); 62 | 42.51 (37.45); 53 | -16.86 (7.20) | -30.98, -2.75 | 0.019 | 0.37 |
| Week 16 | 47.03 (51.37); 60 | 36.97 (35.00); 45 | -9.69 (7.44) | -24.27, 4.88 | 0.192 | 0.21 |
| Week 24 | 44.90 (53.96); 58 | 33.62 (28.36); 46 | -10.81 (7.15) | -24.83, 3.22 | 0.131 | 0.24 |
| **WASO** | | | | | | |
| Baseline | 50.65 (31.73); 65 | 61.78 (69.48); 64 |  |  |  |  |
| Week 10 | 39.57 (36.80); 62 | 25.04 (19.51); 53 | -15.68 (5.63) | -26.72, -4.64 | 0.005 | 0.29 |
| Week 16 | 40.51 (39.11); 60 | 26.18 (23.95); 45 | -16.15 (5.75) | -27.42, -4.90 | 0.005 | 0.30 |
| Week 24 | 43.88 (44.05); 58 | 24.75 (21.60): 46 | 21.60 (6.12) | -33.59, -9.61 | <0.001 | 0.40 |
